# Perioperative Matrix Metalloproteinase-2 and Matrix Metalloproteinase-9 Profiles in Acute and Chronic Subdural Hematomas

**DOI:** 10.3390/jcm15176499

**Published:** 2026-08-22

**Authors:** Bartłomiej Kulesza, Mateusz Krakowiak, Dorota Luchowska-Kocot, Jacek Kurzepa, Cezary Grochowski, Ryszard Maciejewski

**Affiliations:** 1 Institute of Medical Sciences, Faculty of Medicine, John Paul II Catholic University of Lublin, Racławickie Avenue 14, 20-950 Lublin, Poland; 2 Faculty of Medical Sciences and Health Sciences, Radom University, 29 Malczewskiego Street, 26-600 Radom, Poland; 3 Department of Medical Chemistry, Faculty of Medical Sciences, Medical University of Lublin, 20-059 Lublin, Poland; 4 Department of Neurosurgery, Stefan Cardinal Wyszyński Provincial Specialist Hospital Independent Public Health Care Centre, Kraśnicka 100, 20-718 Lublin, Poland

**Keywords:** acute subdural hematoma, chronic subdural hematoma, matrix metalloproteinase-2, matrix metalloproteinase-9, traumatic brain injury

## Abstract

**Background:** Acute subdural hematoma (ASDH) and chronic subdural hematoma (ChSDH) differ substantially in their pathophysiology, clinical course, and outcomes. Matrix metalloproteinases (MMPs), particularly MMP-2 and MMP-9, have been implicated in blood–brain barrier disruption, extracellular matrix remodeling, and inflammatory processes involved in both traumatic brain injury and ChSDH. However, direct comparisons of perioperative MMP profiles between ASDH and ChSDH and their relationships with clinical characteristics remain limited. The aim of this study was to compare perioperative MMP-2 and MMP-9 concentrations in patients with ASDH and ChSDH and explore their associations with selected clinically relevant parameters. **Methods:** Thirty patients undergoing surgical treatment for subdural hematoma were prospectively enrolled, including 10 patients with ASDH and 20 with ChSDH. Serum samples were collected before surgery and on postoperative day 3, and hematoma content was obtained intraoperatively. MMP-2 and MMP-9 concentrations were measured using enzyme-linked immunosorbent assay. **Results:** Patients with ASDH had significantly higher MMP-9 concentrations in hematoma content (*p* = 0.002) and postoperative serum (*p* = 0.029) than patients with ChSDH, whereas MMP-2 concentrations did not differ significantly between the groups. In the ChSDH group, both MMP-2 and MMP-9 concentrations were significantly lower in hematoma content than in preoperative and postoperative serum, while no significant perioperative changes were observed in patients with ASDH. Higher MMP-2 concentrations in hematoma content were associated with lower leukocyte counts and smaller midline shift, whereas higher hematoma MMP-9 concentrations were associated with lower APTT and CRP concentrations after FDR correction. These findings suggest distinct perioperative MMP-2 and MMP-9 profiles in acute and chronic subdural hematomas. **Conclusions:** This study provides a direct comparison of perioperative MMP-2 and MMP-9 profiles in serum and hematoma content between patients with ASDH and ChSDH while also exploring their associations with selected clinically relevant parameters.

## 1. Introduction

Acute subdural hematoma (ASDH) and chronic subdural hematoma (ChSDH) are among the most common intracranial hemorrhages requiring neurosurgical intervention. Although both conditions involve the accumulation of blood within the subdural space, they differ substantially in terms of etiology, pathophysiology, clinical course, and prognosis [1,2]. ASDH most commonly results from severe traumatic brain injury. It represents one of the most serious forms of traumatic intracranial hemorrhage and is frequently associated with extensive primary brain injury. Despite advances in neurosurgical and intensive care management, ASDH continues to be associated with poor outcomes, which are influenced by several clinical and radiological factors [1,3,4].

ChSDH primarily affects older adults and often develops following seemingly minor head trauma. Although ChSDH was first described in 1657, the precise mechanisms underlying its formation and progression remain incompletely understood and continue to be investigated [5]. The development and expansion of ChSDH involve a complex interplay of inflammatory processes, angiogenesis, and disturbances in hemostasis and coagulation [2]. Although surgical treatment of ChSDH is generally considered highly effective, postoperative recurrence remains a major clinical challenge, with reported recurrence rates ranging from 5% to 30% [6].

Matrix metalloproteinases (MMPs) are a family of proteolytic enzymes responsible for the degradation of extracellular matrix components and tissue remodeling. Previous studies have provided evidence that MMPs play an important role in both traumatic brain injury and the pathogenesis of ChSDH [7]. MMP-2 (gelatinase A) and MMP-9 (gelatinase B) belong to the gelatinase subgroup of matrix metalloproteinases. Their primary substrate is type IV collagen, the main structural component of the vascular basement membrane. This makes them key mediators of blood–brain barrier disruption, angiogenesis, and extracellular matrix remodeling [8].

To the best of our knowledge, this is the first study to directly compare MMP-2 and MMP-9 concentrations in patients with acute and chronic subdural hematomas by measuring these biomarkers in preoperative serum, hematoma content, and serum collected three days after surgery, and to explore their associations with selected clinically relevant parameters.

## 2. Patients and Methods

### 2.1. Patients

Patients who underwent surgical treatment for ASDH or ChSDH at the Department of Neurosurgery, Regional Specialist Hospital in Lublin, were eligible for inclusion. The study period was from 1 May 2024 to 1 December 2024. Eight patients were excluded from the analysis due to long-term use of antiplatelet or anticoagulant therapy (*n* = 6) or the presence of concomitant liver or hematological disorders (*n* = 2). Ultimately, 30 patients were included in the study, comprising 10 patients with ASDH and 20 patients with ChSDH.

### 2.2. Sample Collection and MMP Measurements

Peripheral blood samples were collected from each patient 30 min before surgery and again on postoperative day 3. Blood samples were obtained as part of routine clinical care, and no additional blood sampling was performed solely for the purposes of the study. In addition, hematoma content was collected intraoperatively. In patients with ASDH, the hematoma content consisted of freshly evacuated blood clot. In patients with ChSDH, it consisted of liquefied hematoma fluid.

All samples were collected into tubes containing a clot activator and were immediately centrifuged at 2000 rpm for 15 min. After centrifugation, the obtained supernatant was transferred into sterile polypropylene tubes and stored at −80 °C until further analysis. Subsequently, MMP-2 and MMP-9 concentrations were measured using Quantikine ELISA kits (Total MMP-2 Immunoassay, catalog no. MMP200; Human MMP-9 Immunoassay, catalog no. DMP900; R&D Systems, Inc., Minneapolis, MN, USA) at the Department of Medical Chemistry, Medical University of Lublin, Poland.

### 2.3. Analyzed Variables

Baseline demographic, clinical, laboratory, and radiological variables were collected for all patients. The evaluated demographic and clinical parameters included age, sex, admission Glasgow Coma Scale (GCS) score, and in-hospital mortality. Laboratory variables comprised leukocyte count, neutrophil count, lymphocyte count, neutrophil-to-lymphocyte ratio (NLR), hemoglobin concentration, platelet count, platelet-to-lymphocyte ratio (PLR), blood glucose, prothrombin time (PT), activated partial thromboplastin time (APTT), international normalized ratio (INR), C-reactive protein (CRP), and serum creatinine. Radiological variables included hematoma thickness and midline shift (MLS).

Given the limited sample size, the exploratory correlation analysis was restricted to five clinically and biologically relevant variables representing neurological status, inflammation, coagulation, and radiological severity: admission GCS score, leukocyte count, CRP concentration, APTT, and MLS.

### 2.4. Statistical Analysis

Statistical analyses were performed using STATISTICA version 13.0 (StatSoft Polska, Kraków, Poland). The Benjamini–Hochberg false discovery rate (FDR) correction for multiple comparisons was performed using Python version 3.13.5 with the statsmodels package (version 0.14.6). The normality of data distribution was assessed using the Shapiro–Wilk test. Continuous variables are presented as median and interquartile range (IQR), whereas categorical variables are expressed as numbers and percentages.

Comparisons between patients with ASDH and ChSDH were performed using the Mann–Whitney U test for continuous variables and Fisher’s exact test for categorical variables.

As no a priori sample size calculation was performed, a post hoc power analysis was conducted for the main between-group comparison of MMP-9 concentrations in hematoma content. Based on the observed effect size (rank-biserial correlation = 0.875) and a two-sided significance level of α = 0.05, the achieved statistical power for this comparison was approximately 87%.

An exploratory multivariable linear regression analysis was performed to assess whether the between-group difference in hematoma MMP-9 concentrations persisted after adjustment for age, admission GCS score, CRP concentration, and hematoma thickness. Given the limited sample size relative to the number of covariates, this analysis was considered exploratory.

Differences in MMP-2 and MMP-9 concentrations measured before surgery, in hematoma content, and on postoperative day 3 within each study group were analyzed using the Wilcoxon signed-rank test.

Associations between hematoma MMP-2 and MMP-9 concentrations and the selected clinical, laboratory, and radiological variables were assessed using Spearman’s rank correlation coefficient. Given the exploratory nature of the analysis, multiple comparisons were controlled using the Benjamini–Hochberg false discovery rate (FDR) procedure. FDR correction was applied across all 10 correlation tests. Both unadjusted and FDR-adjusted *p*-values are reported. Given the limited sample size, these analyses were considered exploratory and hypothesis-generating.

All statistical tests were two-tailed, and a *p*-value < 0.05 was considered statistically significant.

## 3. Results

### 3.1. Patients and Variables

The ASDH and ChSDH groups differed significantly in several clinical and laboratory parameters. Patients with ASDH presented with more severe neurological impairment on admission and experienced substantially higher mortality than those with ChSDH. In contrast, patients with ChSDH were characterized by greater hematoma thickness and higher CRP concentrations, whereas admission glucose levels were significantly higher in the ASDH group. Platelet count and activated partial thromboplastin time also differed significantly between the groups. No significant between-group differences were observed in demographic characteristics, leukocyte count, neutrophil count, lymphocyte count, NLR, PLR, hemoglobin concentration, PT, INR, creatinine level, or midline shift (Table 1). Postoperative CT was performed in all patients. No clinically significant postoperative rebleeding was identified, and no patient required reoperation before blood sampling on postoperative day 3.

### 3.2. MMP-2 and MMP-9 Concentrations in Patients with ASDH and ChSDH

**Figure 1:**
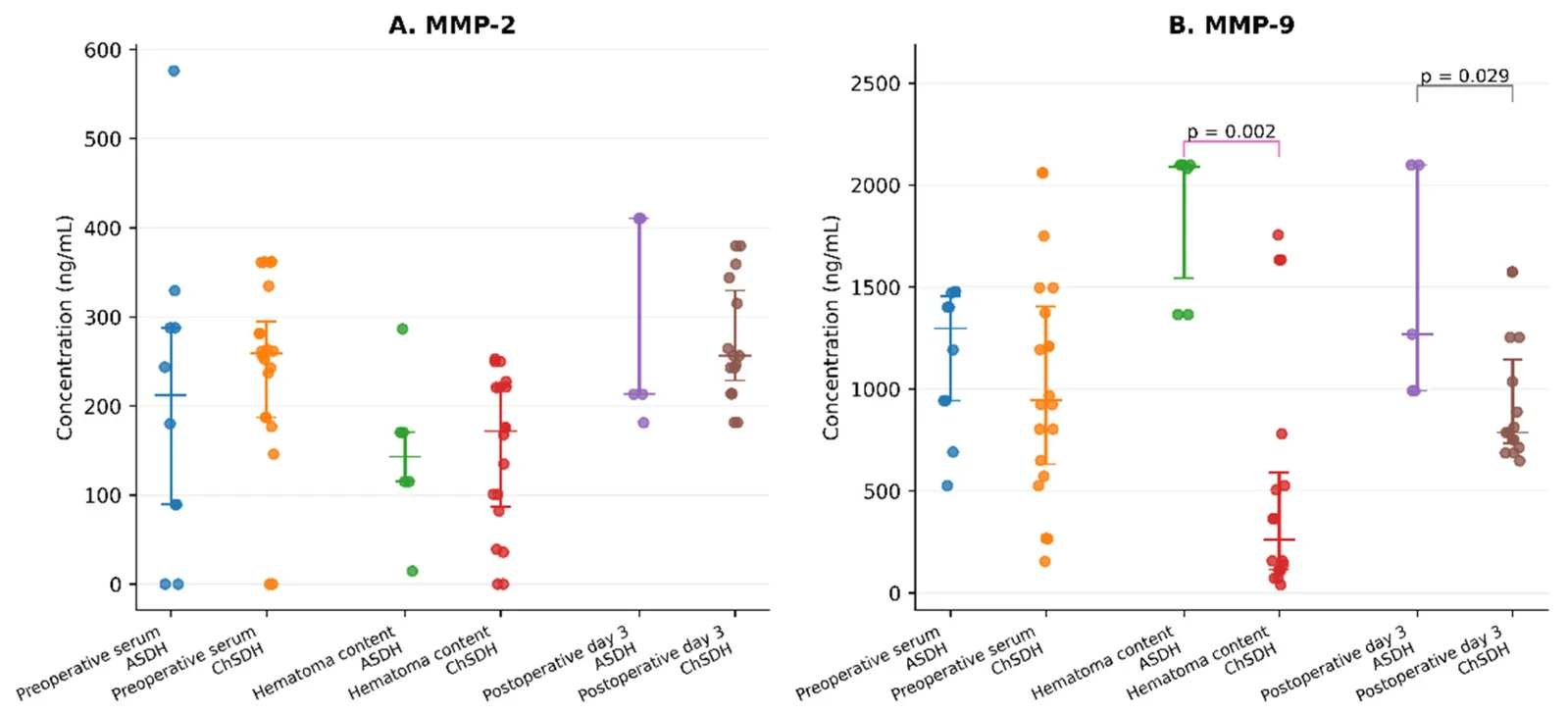
Perioperative MMP-2 and MMP-9 concentrations in patients with acute and chronic subdural hematoma. Individual MMP-2 (**A**) and MMP-9 (**B**) concentrations are shown for patients with acute subdural hematoma (ASDH) and chronic subdural hematoma (ChSDH) in preoperative serum, hematoma content, and serum collected on postoperative day 3. Points represent individual measurements, and summary bars represent the median and interquartile range. Between-group comparisons were performed using the Mann–Whitney U test. Significant differences are indicated by the corresponding *p*-values.

No significant differences were observed between the ASDH and ChSDH groups with respect to MMP-2 concentrations at any sampling time point. Likewise, preoperative serum MMP-9 concentrations were comparable between the groups. In contrast, MMP-9 concentrations in blood clot and on postoperative day 3 were significantly higher in patients with ASDH (Table 2, Figure 1). In an exploratory multivariable analysis adjusting for age, admission GCS score, CRP concentration, and hematoma thickness, the between-group difference in hematoma MMP-9 concentration was no longer statistically significant (β = 529.4, 95% CI −1119.4 to 2178.3; *p* = 0.502).

No significant changes in either MMP-2 or MMP-9 concentrations were observed across the three sampling time points in patients with ASDH. In contrast, significant temporal differences were found in patients with ChSDH for both MMP-2 and MMP-9. In this group, concentrations of both metalloproteinases were significantly lower in hematoma content than in serum obtained before surgery and on postoperative day 3, whereas no significant differences were observed between the two serum measurements (Table 3).

### 3.3. Correlation Analysis Between Hematoma MMP-2 and MMP-9 Concentrations and Selected Variables

Correlation analyses were restricted to five clinically and biologically relevant variables. Given the limited sample size, these correlation analyses should be considered exploratory and hypothesis-generating. After correction for multiple comparisons using the Benjamini–Hochberg FDR procedure, higher MMP-2 concentrations in hematoma content were significantly associated with lower leukocyte count (ρ = −0.710, adjusted *p* = 0.001) and lower midline shift (ρ = −0.497, adjusted *p* = 0.036). Higher MMP-9 concentrations in hematoma content were significantly associated with lower APTT (ρ = −0.514, adjusted *p* = 0.036) and lower CRP concentrations (ρ = −0.541, adjusted *p* = 0.036). No other correlations remained statistically significant after FDR correction (Table 4).

## 4. Discussion

The distinct MMP-9 profiles observed in ASDH and ChSDH may reflect differences in the biological processes underlying acute traumatic injury and chronic hematoma development. Previous studies have shown that MMP-9 is involved in the acute inflammatory response following traumatic brain injury and may contribute to blood–brain barrier disruption, extracellular matrix degradation, cerebral edema, and secondary brain injury [9,10]. In contrast, chronic subdural hematoma is characterized by a gradually developing inflammatory and angiogenic response, with local accumulation of inflammatory mediators [2,11]. These differences provide a possible biological context for the higher MMP-9 concentrations observed in patients with ASDH. However, as specific inflammatory mediators and biomarkers of brain injury were not measured in our study, our findings do not provide direct evidence of an association between MMP-9, inflammation, and secondary brain injury. However, the between-group difference in hematoma MMP-9 was no longer statistically significant after adjustment for age, GCS, CRP, and hematoma thickness. Given the small sample size and the resulting imprecision of the multivariable model, this finding does not allow firm conclusions regarding whether the observed difference in MMP-9 is independent of differences in clinical severity between ASDH and ChSDH.

MMP-9 concentrations in both cerebrospinal fluid and blood increase within the first hours after severe traumatic brain injury. Higher levels are associated with more extensive brain damage and a poorer prognosis [12]. Furthermore, elevated MMP-9 concentrations have been shown to correlate with lower Glasgow Coma Scale scores, more severe cerebral edema, higher mortality, and worse neurological outcomes [12]. The inverse association with CRP does not support a simple positive relationship between local MMP-9 concentrations and systemic inflammation. CRP is a nonspecific systemic inflammatory marker and may not directly reflect local processes within the subdural compartment. However, given the limited sample size and exploratory nature of the analysis, these findings should be interpreted cautiously.

The relatively similar MMP-2 profiles in ASDH and ChSDH may reflect the different biological roles of MMP-2 compared with MMP-9. Given the limited sample size, the absence of statistically significant between-group differences in MMP-2 concentrations should not be interpreted as evidence of equivalence between the groups. This finding may be considered in the context of the biological role of MMP-2, which is primarily involved in extracellular matrix remodeling, tissue repair, and angiogenesis rather than in the acute inflammatory response following traumatic brain injury. Unlike MMP-9, whose expression is rapidly induced after injury, MMP-2 shows no marked early increase and instead contributes predominantly to the later stages of tissue remodeling and vascular maturation [13,14]. This biological profile may account for the relatively stable MMP-2 concentrations observed in both ASDH and ChSDH.

Previous studies have suggested that MMP-2 may be involved in tissue repair and remodeling after brain injury [14]. Higher MMP-2 concentrations have also been associated with lower leukocyte counts, less severe cerebral edema, higher Glasgow Coma Scale scores, and more favorable neurological outcomes [15]. The associations observed in our study between hematoma MMP-2 concentrations, leukocyte counts, and midline shift may therefore reflect a relationship between MMP-2 and the biological and radiological characteristics of brain injury. However, these findings should not be interpreted as evidence of a protective effect of MMP-2. Given the observational design and limited sample size, they should be considered hypothesis-generating and require validation in larger prospective studies.

The lower MMP-2 and MMP-9 concentrations observed in ChSDH hematoma content relative to serum warrant consideration in the context of previous studies. Similar findings for MMP-9 have been reported in previous studies. Su et al. found lower MMP-9 concentrations in hematoma fluid than in preoperative and postoperative serum in patients with ChSDH [16]. Edlmann et al. also reported lower MMP-9 concentrations in ChSDH fluid than in plasma [17]. However, the results for MMP-2 are less consistent. Hua et al. reported higher MMP-2 concentrations in hematoma fluid than in serum [7], which is different from our findings. Nakagawa et al. also showed increased MMP expression and activity in chronic subdural hematoma membranes [18]. These differences may be related to the biological material examined, patient characteristics, and the methods used. The hematoma membrane and hematoma fluid represent different biological compartments. The membrane is an active tissue involved in inflammation, angiogenesis, and vascular remodeling. Therefore, increased MMP expression or activity in the membrane does not necessarily mean that MMP concentrations are also high in the hematoma fluid. In our study, we measured total MMP-2 and MMP-9 concentrations in liquefied hematoma content using ELISA. We did not measure MMP expression or enzymatic activity in the hematoma membrane. This may partly explain the differences between our results and those of previous studies.

Although the present findings do not support the routine clinical use of MMP-2 or MMP-9 measurements, previous studies suggest a potential prognostic role for MMP-9 after traumatic brain injury. Copin et al. reported that elevated early plasma MMP-9 concentrations were associated with an increased risk of prolonged ICU stay or death after severe TBI [19]. Similarly, de Lima et al. found that higher plasma MMP-9 concentrations were independently associated with ICU mortality, whereas MMP-2 concentrations were not associated with mortality [20]. These findings suggest that MMP-9 may provide additional information on the biological severity of acute brain injury and could potentially complement established clinical and radiological prognostic factors. Serial measurements may also help characterize changes in the biological response during the perioperative period. However, the prognostic value of MMP-9 remains uncertain, and our study was not designed to determine whether MMP measurements improve prognostic accuracy, guide treatment selection, or influence patient monitoring. Larger prospective studies are required to determine whether MMP-9 provides additional prognostic value beyond established clinical and radiological predictors and whether it could contribute to clinical decision-making.

## 5. Limitation

This study has several limitations. The sample size was small, particularly in the ASDH group, which limited statistical power. Therefore, the correlation analyses were restricted to selected clinically relevant variables and should be considered exploratory and hypothesis-generating. Although the Benjamini–Hochberg FDR correction was applied, these findings require confirmation in larger cohorts. The single-center design further limits the generalizability of the results.

Patients receiving long-term antiplatelet or anticoagulant therapy were excluded, which may have introduced selection bias, particularly in the ChSDH group, where antithrombotic therapy is common. In addition, only surgically treated patients were included because intraoperative hematoma sampling was required. Therefore, our findings may not fully represent routine clinical practice and should not be generalized to conservatively treated patients.

Only total MMP-2 and MMP-9 concentrations were measured, without assessment of enzymatic activity, membrane tissue expression, or TIMP-1 and TIMP-2. Since MMP activity depends partly on the MMP/TIMP balance, measured concentrations should not be interpreted as direct evidence of proteolytic activity. The different physical composition of freshly evacuated ASDH clot and liquefied ChSDH fluid may also have affected biomarker recovery and comparability between the two sample types. Specific inflammatory mediators and biomarkers of brain injury were not assessed, which further limits the mechanistic interpretation of the observed MMP profiles.

Detailed measures of traumatic brain injury severity, including cerebral contusions, diffuse axonal injury, pupillary status, and Injury Severity Score, were not systematically collected. Similarly, the time from injury to surgery in ASDH and symptom duration before surgery in ChSDH were not systematically recorded. These factors may have influenced the observed MMP concentrations. Finally, postoperative infections and seizures were not systematically recorded, and their potential effect on MMP concentrations measured on postoperative day 3 cannot be excluded.

## 6. Conclusions

Patients with ASDH had significantly higher MMP-9 concentrations in hematoma content than patients with ChSDH. Postoperative serum MMP-9 concentrations on day 3 were also higher in the ASDH group. In contrast, MMP-2 concentrations did not differ significantly between the groups. These findings suggest distinct perioperative MMP profiles in ASDH and ChSDH.

Higher MMP-2 concentrations in hematoma content were associated with lower leukocyte counts and smaller midline shift, whereas higher hematoma MMP-9 concentrations were associated with lower APTT and CRP concentrations after FDR correction. Given the limited sample size, these associations should be considered exploratory and require confirmation in larger studies.

Further larger prospective studies evaluating MMP concentrations and enzymatic activity, together with tissue inhibitors of metalloproteinases and specific inflammatory and brain injury biomarkers, are needed to better define their pathophysiological and prognostic significance.

## Figures and Tables

**Table 1:** Baseline characteristics of patients with ASDH and ChSDH.

Variable	ChSDH (*n* = 20)	ASDH (*n* = 10)	*p*-Value
Demographic characteristics			
Age, years	72.5 (62.0–81.2)	54.0 (47.0–80.0)	0.165
Male sex, *n* (%)	15 (75.0)	6 (60.0)	0.638
Clinical characteristics			
Glasgow Coma Scale	**13.0 (11.8–15.0)**	**8.5 (4.2–12.0)**	**0.019**
Mortality, *n* (%)	**4 (20.0)**	**8 (80.0)**	**0.005**
Survival, *n* (%)	16 (80.0)	2 (20.0)	—
Laboratory parameters			
Leukocyte count (×10^9^/L)	7.9 (6.1–12.0)	10.0 (8.3–13.2)	0.179
Neutrophil count (×10^9^/L)	8.4 (4.7–10.0)	7.4 (5.3–9.1)	0.913
Lymphocyte count (×10^9^/L)	1.0 (0.4–2.5)	1.3 (0.9–1.4)	0.856
Neutrophil-to-lymphocyte ratio	5.3 (4.0–6.7)	5.5 (3.2–9.2)	0.971
Hemoglobin (g/dL)	12.8 (11.9–13.8)	12.4 (10.6–13.3)	0.378
Platelet count (×10^9^/L)	**216.0 (195.5–247.0)**	**174.0 (152.0–225.5)**	**0.045**
Platelet-to-lymphocyte ratio	175.8 (134.1–287.6)	178.9 (145.1–197.4)	0.637
Glucose (mg/dL)	**111.0 (90.0–129.0)**	**161.0 (141.5–171.0)**	**0.008**
PT (s)	12.2 (12.0–12.9)	12.4 (11.8–13.0)	0.184
APTT (s)	**29.9 (27.8–34.3)**	**25.7 (24.7–28.5)**	**0.019**
INR	1.05 (1.03–1.11)	1.05 (1.02–1.08)	0.126
CRP (mg/L)	**8.8 (3.0–14.2)**	**1.8 (1.1–2.1)**	**0.023**
Creatinine (mg/dL)	0.99 (0.82–1.18)	0.83 (0.58–1.06)	0.186
Radiological findings			
Hematoma thickness (mm)	**20.5 (20.0–25.0)**	**9.5 (7.0–20.0)**	**0.034**
Midline shift (mm)	5.5 (2.3–10.0)	10.0 (4.5–22.5)	0.137

Data are presented as median (interquartile range) unless otherwise indicated. Categorical variables are presented as number (%). Continuous variables were compared using the Mann–Whitney U test and categorical variables using Fisher’s exact test. Statistically significant differences (*p* < 0.05) are shown in bold.

**Table 2:** Comparison of MMP-2 and MMP-9 concentrations between patients with ASDH and ChSDH.

Biomarker	ChSDH (*n* = 20)	ASDH (*n* = 10)	*p*-Value
MMP-2			
Before surgery (ng/mL)	258.9 (187.1–294.5)	211.8 (89.5–287.8)	0.467
Hematoma content (ng/mL)	171.8 (86.8–221.2)	142.9 (115.3–170.5)	0.920
Postoperative day 3 (ng/mL)	256.6 (228.5–329.5)	213.1 (213.1–410.6)	0.793
MMP-9			
Before surgery (ng/mL)	945.6 (631.0–1403.9)	1296.8 (943.0–1454.3)	0.509
Hematoma content (ng/mL)	**261.3 (113.8–590.2)**	**2089.5 (1544.1–2098.4)**	**0.002**
Postoperative day 3 (ng/mL)	**787.0 (732.7–1145.2)**	**1268.1 (991.0–2098.4)**	**0.029**

Data are presented as median (interquartile range). Comparisons between groups were performed using the Mann–Whitney U test. Statistically significant differences (*p* < 0.05) are shown in bold.

**Table 3:** Within-group comparisons of perioperative MMP-2 and MMP-9 concentrations in patients with ASDH and ChSDH.

Comparison	ChSDH, *p*-Value	ASDH, *p*-Value
MMP-2		
Before surgery vs. hematoma content	**0.003**	0.469
Before surgery vs. POD3	0.460	0.750
Hematoma content vs. POD3	**0.004**	0.500
MMP-9		
Before surgery vs. hematoma content	**<0.001**	0.125
Before surgery vs. POD3	0.394	0.313
Hematoma content vs. POD3	**0.002**	1.000

Values represent *p*-values from within-group pairwise comparisons performed using the Wilcoxon signed-rank test. Statistically significant differences (*p* < 0.05) are shown in bold. POD3, postoperative day 3.

**Table 4:** Correlations between MMP-2 and MMP-9 concentrations in hematoma content and selected clinically relevant variables.

Variable	MMP-2 ρ	*p*-Value	FDR-Adjusted *p*-Value	MMP-9 ρ	*p*-Value	FDR-Adjusted *p*-Value
Glasgow Coma Scale	0.393	0.057	0.115	−0.380	0.081	0.136
Leukocyte count	−0.710	<0.001	**0.001**	0.179	0.425	0.472
APTT	0.143	0.504	0.504	−0.514	0.014	**0.036**
CRP	0.274	0.217	0.271	−0.541	0.014	**0.036**
Midline shift	−0.497	0.013	**0.036**	0.323	0.143	0.204

ρ, Spearman’s rank correlation coefficient; APTT, activated partial thromboplastin time; CRP, C-reactive protein. *p*-values were adjusted for multiple comparisons using the Benjamini–Hochberg false discovery rate (FDR) procedure across all 10 correlation tests. Statistically significant results after FDR correction (adjusted *p* < 0.05) are shown in bold.

## Data Availability

The data generated and analyzed during this study are not publicly available due to privacy considerations related to patient data.
